# APPL proteins promote TGFβ-induced nuclear transport of the TGFβ type I receptor intracellular domain

**DOI:** 10.18632/oncotarget.6346

**Published:** 2015-11-18

**Authors:** Jie Song, Yabing Mu, Chunyan Li, Anders Bergh, Marta Miaczynska, Carl-Henrik Heldin, Marene Landström

**Affiliations:** ^1^ Medical Biosciences, Umeå University, Umeå, Sweden; ^2^ Implant Center, Stomatological Hospital, Jilin University, Changchun, China; ^3^ International Institute of Molecular and Cell Biology, Laboratory of Cell Biology, Warsaw, Poland; ^4^ Ludwig Institute for Cancer Research Ltd, Science for Life Laboratory, Uppsala University, Uppsala, Sweden

**Keywords:** APPL proteins, prostate cancer, signal transduction, tumour necrosis factor receptor-associated factor 6, transforming growth factor β

## Abstract

The multifunctional cytokine transforming growth factor-β (TGFβ) is produced by several types of cancers, including prostate cancer, and promote tumour progression in autocrine and paracrine manners. In response to ligand binding, the TGFβ type I receptor (TβRI) activates Smad and non-Smad signalling pathways. The ubiquitin-ligase tumour necrosis factor receptor-associated factor 6 (TRAF6) was recently linked to regulate intramembrane proteolytic cleavage of the TβRI in cancer cells. Subsequently, the intracellular domain (ICD) of TβRI enters in an unknown manner into the nucleus, where it promotes the transcription of pro-invasive genes, such as *MMP2* and *MMP9*. Here we show that the endocytic adaptor molecules APPL1 and APPL2 are required for TGFβ-induced nuclear translocation of TβRI-ICD and for cancer cell invasiveness of human prostate and breast cancer cell lines. Moreover, APPL proteins were found to be expressed at high levels in aggressive prostate cancer tissues, and to be associated with TβRI in a TRAF6-dependent manner. Our results suggest that the APPL–TβRI complex promotes prostate tumour progression, and may serve as a prognostic marker.

## INTRODUCTION

Prostate cancer is the second most common cause of cancer-related death among men in the Western world [1]. High levels of transforming growth factor β (TGFβ) is known to be associated with a poor prognosis for the patients [2]. Members of the TGFβ family regulate cell proliferation, differentiation, apoptosis, migration and the epithelial-mesenchymal transition (EMT), and are therefore important for embryogenesis and tissue homeostasis in adults, as well as for cancer progression. TGFβ signals by binding to type I and type II serine/threonine kinase receptors (TβRI and TβRII, respectively). After ligand-induced formation of heterotetrameric complexes of TβRII and TβRI, TβRII phosphorylates the glycine-serine (GS)-rich domain of TβRI, leading to conformational changes and activation of the kinase activity of TβRI. Activated TβRI induces phosphorylation of the C-terminal SSXS motif of receptor-activated SMADs (SMAD2 and 3), which then translocate to the nucleus where they regulate the expression of target genes together with the common mediator SMAD4 [3–7]. TGFβ also signals via non-SMAD pathways, including Erk, JNK and p38 MAP-kinase pathways, phosphatidylinositol 3-kinase (PI3K) and the tyrosine kinase Src [8, 9]. For activation of the TGFβ-activated kinase 1 (TAK1) and the downstream p38/JNK pathways that promote apoptosis and EMT, the tumour necrosis factor-associated factor 6 (TRAF6) is essential [10, 11]. TRAF6 also promotes the proteolytic cleavage of TβRI and the release of the intracellular domain (ICD) that translocates to the nucleus in an unknown manner, and drives an invasiveness programme [12, 13].

TGFβ receptor signalling is regulated by both clathrin-mediated endocytosis, which is used by many cell-surface receptors such as the epidermal growth factor (EGF) receptor and G protein-coupled receptors, as well as by lipid raft/caveolin-mediated endocytosis [14]. In clathrin-dependent endocytosis, TGFβ receptors are internalized to phosphatidylinositol-3-phosphate (PI3P)-enriched, early endosome antigen-1 (EEA1)-positive endosomes, where they interact with the SMAD anchor for receptor activation (SARA) to induce the canonical SMAD-dependent signalling pathway [15]. From the early endosome, TGFβ receptors can be recycled back to the cell surface in the absence of ligand by entering Rab11-positive endosomes [16, 17]. In lipid raft/caveolae-dependent endocytosis, the association of TGFβ receptors with SMAD7 and the ubiquitin ligase Smurf2 facilitates the ubiquitin-dependent degradation of the receptors by their internalization into caveolin-1-positive vesicles [15].

The small GTPase Rab5 is a key regulator of the early steps of endosomal sorting and is implicated in transducing TGFβ signals [18]. Rab5 interacts with and regulates the activities of EEA1 and PI3K [19, 20]. APPL1 and APPL2 are Rab5 effector proteins and are multifunctional adaptor proteins that contain an N-terminal bin1/amphiphysin/Rvs 167 (BAR) domain, a pleckstrin homology (PH) domain, and a C-terminal phosphotyrosine binding (PTB) domain [21]. APPL endosomes can act as precursors of classical EEA1-positive endosomes, and depletion of PI-3-phosphate leads to an expansion of the APPL compartments [22]. APPL1 is implicated in several signalling pathways, such as the EGF [23], androgen [24], and NF-κB [25] pathways, but has not previously been linked to TGFβ signalling. Upon EGF stimulation, APPL1 is released from early endosomes and translocates to the nucleus, where it associates with the nucleosome remodelling and histone deacetylation machinery [23, 26, 27]. In addition, APPL1 interacts with signalling proteins, including Akt [21] and PI3K [21, 24]. Through its varied interactions, APPL1 has been reported to mediate apoptosis [28], cell proliferation [23, 26], as well as the endosomal localization of proteins [29, 30]. APPL1 and Rab5a have recently been found to be overexpressed in lung adenocarcinoma [31], but the precise role of APPL proteins in malignancies is still unknown.

To elucidate the molecular mechanisms mediating invasion of prostate cancer cells, we investigated the role of APPL proteins in TGFβ-induced signal transduction. In this report, we identify APPL1 as a TβRI- and PKCζ-associated protein and show that APPL1 and APPL2 are required for the nuclear translocation of TβRI-ICD, and thereby promote progression of prostate cancer cells.

## RESULTS

### Nuclear accumulation of the ICD of TβRI is dependent on APPL proteins

We have previously reported that TβRI undergoes proteolytic cleavage in cancer cells, whereby TβRI-ICD is released from the membrane and is translocated into the nucleus, however, the molecular mechanism involved is not known [12, 13]. As APPL proteins act as adaptor proteins that facilitate cargo trafficking from the endosomal membranes to the nucleus in response to EGF [24, 32], we explored the possibility that APPL proteins are involved in the TGFβ-induced nuclear translocation of TβRI-ICD.

**Figure 1:**
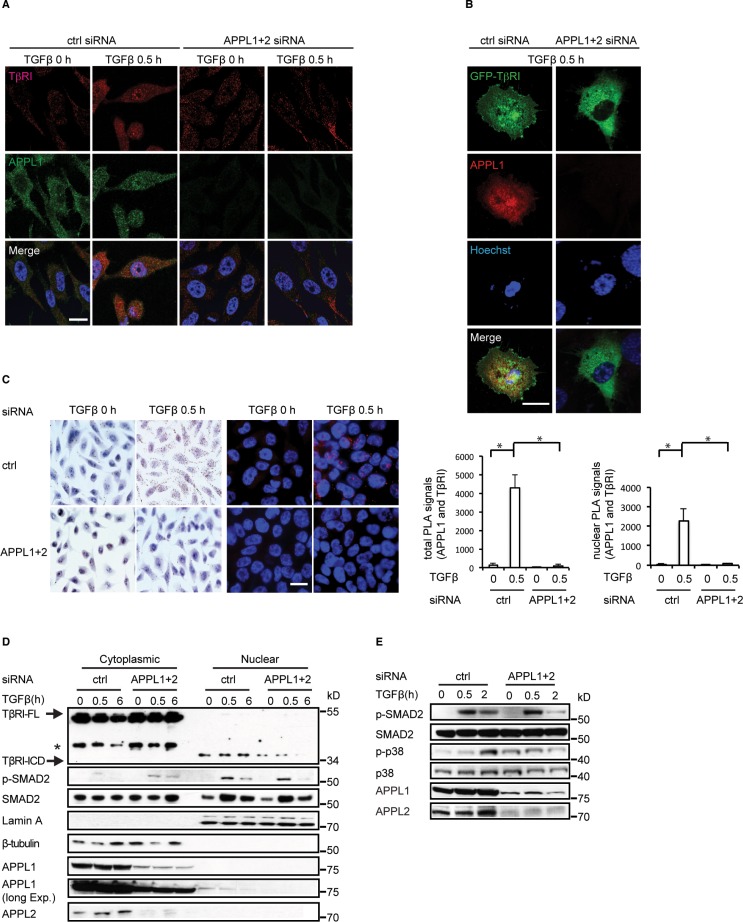
APPL proteins are involved in the nuclear translocation of TβRI-ICD and in TGFβ signalling (**A**) PC-3U cells in which APPL1 and APPL2 expression was, or was not, silenced using siRNA were treated with TGFβ. APPL1 and TβRI were visualized by immunofluorescence using APPL1 and V22 (FITC) antisera. Scale bar, 20 μm. (**B**) C-terminally GFP-tagged TβRI was visualized in TGFβ-stimulated cells treated, or not, with APPL1 and APPL2 siRNA. (**C**) PLA analysis (brightfield and immunofluorescence) of TGFβ-induced formation of APPL1-TβRI-ICD complex (brown and red, respectively). Quantification of total and nuclear PLA signals are shown in the right panel (mean ± SD of three experiments; 350 cells were analyzed in each group). (**D**) Expression of APPL1 and APPL2 in PC-3U cells was knocked down using siRNA. The cells were subjected to cytoplasmic and nuclear protein fractionation; the lysates were analyzed by SDS-gel electrophoresis followed by immunoblotting for TβRI-ICD and p-SMAD2. Lamin A and β-tubulin served as controls for the nuclear and cytoplasmic fractions, respectively. Asterisk marks a background band. (**E**) PC-3U cells in which APPL1 and APPL2 expression was or was not silenced by siRNA were treated with TGFβ, or not, and subjected to immunoblotting for p-p38 and p-SMAD2.

We investigated the subcellular localization of endogenous APPL1 and TβRI-ICD in human prostate cancer PC-3U cells using immunofluorescence staining and confocal imaging. Interestingly, stimulation by TGFβ for 30 minutes induced the co-localization of APPL1 and TβRI-ICD in the nucleus, as visualized by staining with antibodies recognizing APPL1 and the C-terminal part of TβRI, respectively [12, 23]. By contrast, almost no TβRI-ICD translocated into the nuclei of TGFβ-treated cells after knockdown of endogenous APPL1 and APPL2 (Figure 1A). Next, we analyzed the localization of a fusion protein in which GFP was linked to the C-terminus of TβRI-ICD. GFP-TβRI-ICD co-localized with endogenous APPL1 in PC-3U nuclei after TGFβ treatment for 30 minutes, whereas there was no significant nuclear accumulation of GFP-TβRI-ICD when APPL1 and APPL2 expression was silenced (Figure 1B). To further explore the possibility that TGFβ induces a complex between APPL1 and TβRI-ICD, we performed a proximity ligation assay (PLA) [33]. We found that TGFβ stimulation induced a complex of APPL1 and TβRI in cells, as shown by the red dots in Figure 1C; as expected, no such signals were seen when APPL1/2 was knocked down by siRNA (Figure 1C).

To further confirm that APPL proteins participate in the nuclear translocation of TβRI-ICD, we performed nuclear fractionation of cell lysates from TGFβ-treated PC-3U cells. We observed that nuclear accumulation of endogenous TβRI-ICD decreased after siRNA-mediated silencing of APPL1 and APPL2 (Figure 1D). In addition, knockdown of APPL1 and APPL2 led to less accumulation of a HA-tagged TβRI-ICD [13] in the nucleus (Figure S1A). That APPL1 promotes TGFβ-induced translocation of TβRI-ICD is further supported by the observation that transient overexpression of GFP-APPL1 enhanced the nuclear translocation of TβRI-ICD (Figure S1B). The amount of nuclear p-SMAD2 decreased slightly at 2–6 hours after TGFβ stimulation of APPL1- and APPL2-depleted cells (Figure 1D, 1E). Moreover, the level of p-p38 was increased in APPL1 and APPL2- silenced cells, but did not increase significantly in response to TGFβ stimulation (Figure 1E). These findings suggest that APPL proteins are implicated in TGFβ-stimulated p38 MAPK and SMAD2 activation, as well as in the nuclear accumulation of TβRI-ICD in PC-3U cells.

### APPL forms a complex with TβRI

**Figure 2:**
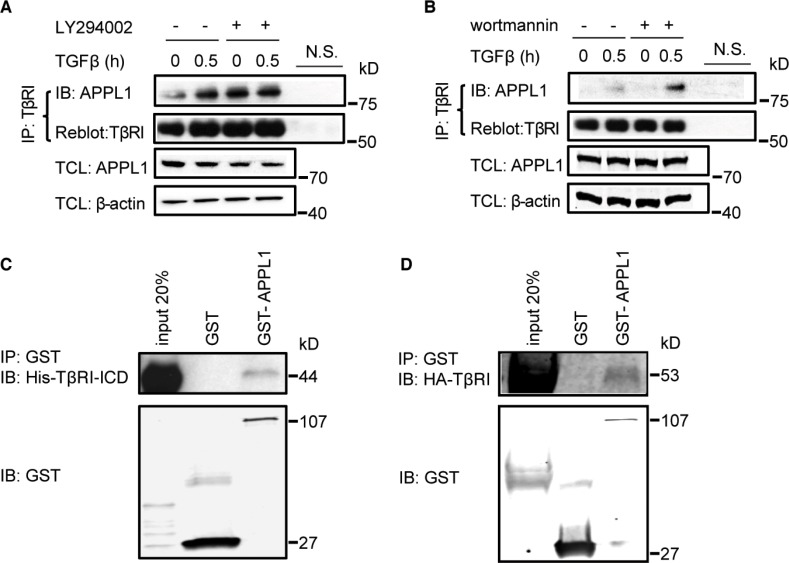
APPL1 associates with TβRI (**A**), (**B**) Inhibition of the activity of PI3 kinase enhanced the association between endogenous TβRI and APPL1. Cell lysates from PC-3U cells treated or not with TGFβ and the PI3 kinase inhibitors LY294002 or wortmannin, were immunoprecipitated with an antibody against TβRI (V22) and subjected to immunoblotting with an APPL1 antibody. N.S., non-specific control, including beads control and IgG control. (**C**), (**D**) *In vitro* interaction of GST-APPL1 proteins, with His-TβRI-ICD and HA-TβRI.

APPL endosomes have been found to be crucial for cargo trafficking and signal transduction downstream of various receptors including adiponectin receptors [32]. APPL endosomes are thought to be the precursors of classical EEA1-positive endosomes, and PI3P is important for their maturation [22]. We therefore investigated whether APPL1 forms a physical complex with TβRI. We performed co-immunoprecipitation experiments using lysates of PC-3U cells that were treated, or not, with TGFβ. We observed that TGFβ treatment promoted an interaction between endogenous APPL1 and the full length TβRI (Figure 2A, 2B). Notably, this interaction was enhanced when the cells were pretreated with the PI3K inhibitors LY294002 or wortmannin, which prevent the maturation of APPL-positive endosomes into EEA1-positive endosomes (Figure 2A, 2B). By contrast, treatment of cells with the TβRI kinase inhibitor SB 505124 had no effect on the interaction of APPL1 and TβRI (Figure S1C). Moreover, His-tagged TβRI-ICD and HA-TβRI bound to recombinant GST-tagged APPL1 (Figure 2C, 2D), confirming the results shown in Figure 2A, 2B, that endogenous proteins interacted.

To elucidate which part of APPL1 mediates binding to TβRI, we performed co-immunoprecipitation and immunofluorescence experiments with various APPL fragments. Transient expression of HA-TβRI together with two different GFP-APPL truncation mutants in PC-3U cells revealed that the C-terminus, but not the N-terminus, of APPL1 bound to TβRI (Figure S1D, S1E). We conclude that TGFβ promotes the association of TβRI and APPL1, consistent with the possibility that at least a portion of TβRI is found in APPL1-positive endosomes.

### TRAF6 is required for TβRI–APPL complex formation

**Figure 3:**
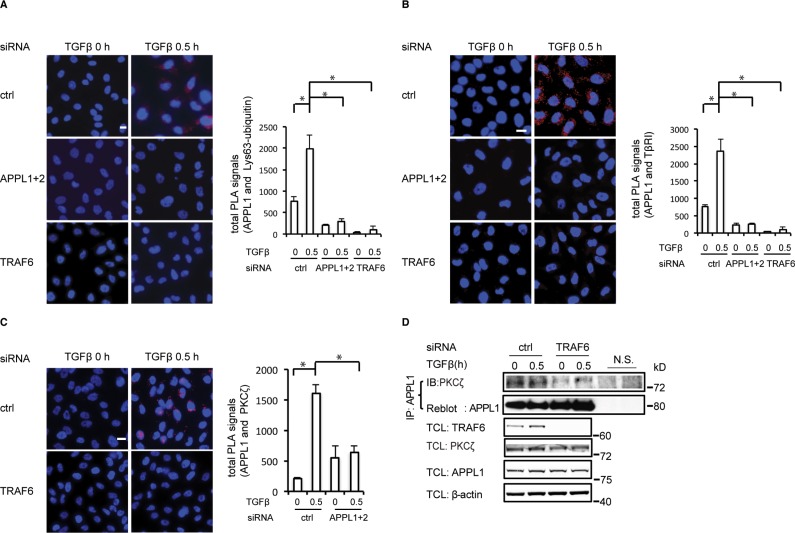
TRAF6 promotes the formation of the TβRI-APPL1 complex (**A**) Representative images showing the association between APPL1 and Lys63-polyubiquitin (red), determined by PLA in PC-3U cells in which TRAF6 or APPL proteins were knocked down, or not, in the presence or absence of TGFβ. (**B**) Association between APPL1 and TβRI (red), determined by PLA in PC-3U cells in which TRAF6 or APPL proteins were knocked down, or not. (**C**) PLA was used to determine the formation of a complex between APPL1 and PKCζ in PC-3U cells in which TRAF6 proteins were silenced, or not. Quantifications of total and nuclear PLA signals in the right panel show the means ± SD of three experiments; 350 cells were analyzed in each group (in Figure 3A–3C). (**D**) Silencing the expression of TRAF6 decreased the association between endogenous PKCζ and APPL1. Cell lysates from PC-3U cells treated as indicated were immunoprecipitated with an antibody against APPL1 and subjected to immunoblotting with a PKCζ antibody.

The subcellular localization of proteins can be modulated by post-translational modifications, such as phosphorylation and Lys63-linked polyubiquitination [34]. We have previously shown that the E3 ubiquitin ligase TRAF6 is required for the Lys63-linked polyubiquitination of TβRI and the subsequent formation of the TβRI- ICD in response to stimulation with TGFβ, in a manner dependent on PKCζ [10]. We therefore investigated the role of TRAF6 in TGFβ-induced endosomal sorting of TβRI to APPL1-positive endosomes. PLA analysis using antisera against Lys63-linked polyubiquitin and APPL1 suggested that APPL1 is subjected to Lys63-linked polyubiquitination (Figure 3A). Moreover, TβRI was sorted to APPL1-positive endosomes in control cells, but not in cells depleted of TRAF6 by siRNA (Figure 3B). As we have previously shown that TRAF6 and PKCζ are important for proteolytic cleavage of TβRI, we investigated whether APPL1 interacts with PKCζ. A TGFβ-induced association of APPL1 with PKCζ was demonstrated by PLA analysis (Figure 3C); this interaction was not seen after knockdown of TRAF6 by siRNA, as analyzed by co-immunoprecipitation (Figure 3D). PKCζ pseudosubstrate or TβRI kinase inhibitors had no effect on the association between APPL1 and PKCζ (Figure S2A, S2B). These observations suggest that TRAF6 promotes the sorting of TβRI to APPL1-positive endosomes.

### APPL proteins are involved in the trafficking of TβRI-ICD to the nucleus

**Figure 4:**
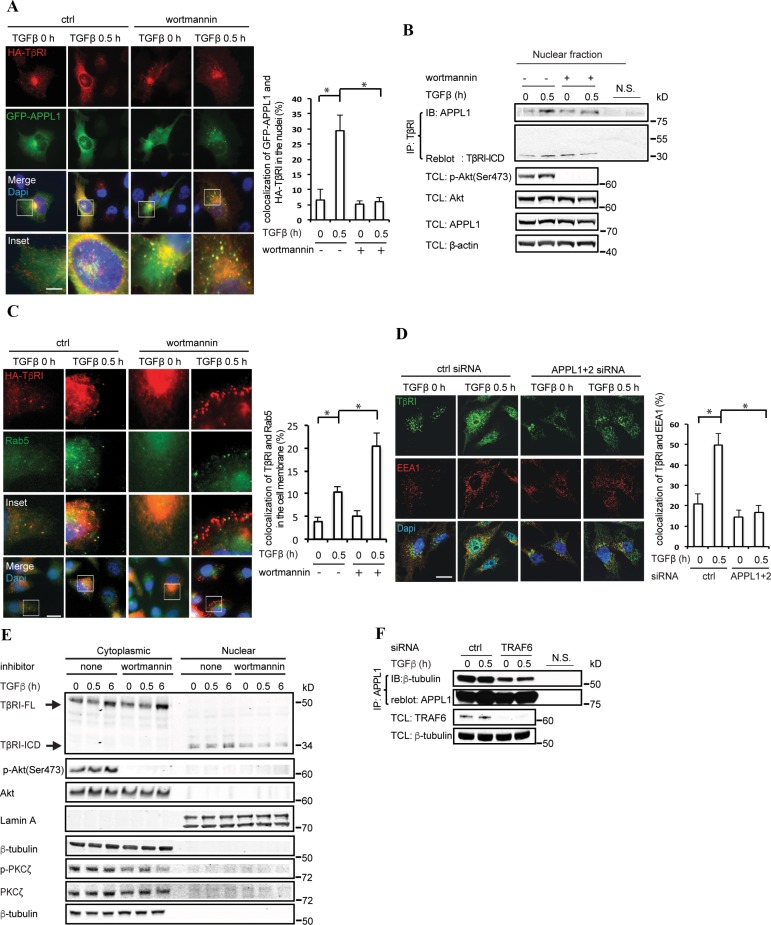
APPL proteins are necessary for trafficking of TβRI-ICD from the cell membrane to the nucleus (**A**) PC-3U cells transiently transfected with GFP-APPL1 and C-terminally tagged HA-TβRI were treated, or not, with TGFβ and wortmannin. Protein co-localization is shown as yellow dots in the merged images. Bar graph shows the percentage of GFP-APPL1 and HA-TβRI complex in the nuclei, based on 300 cells counted in each group. Scale bar, 20 μm. (**B**) The PC-3U cells were treated with wortmannin as indicated and then subjected to cytoplasmic and nuclear protein fractionation, and the nuclear lysates were immunoprecipitated with TβRI antibody (V22) followed by immunoblotting with APPL1 antibody. (**C**) PC-3U cells transiently transfected with HA-TβRI were untreated or treated with TGFβ and wortmannin and then stained with HA and Rab5 antibodies. Bar graph shows the percentage of TβRI-Rab5 complex in the cell membrane, based on 300 cells counted in each group. Scale bar, 20 μm. (**D**) Confocal immunofluorescence analysis of EEA1 and TβRI was performed in PC-3U cells in which APPL1 and APPL2 expression was, or was not, silenced by siRNA. Scale bar, 20 μm. Bar graph shows the percentage of TβRI-EEA1 complex in PC-3U cells, based on 300 cells counted in each group. Scale bar, 20 μm. (**E**) PC-3U cells, which were treated with wortmannin, were subjected to cytoplasmic and nuclear protein fractionation, and the lysates were subjected to SDS-gel electrophoresis, followed by immunoblotting to detect TβRI-ICD, Akt and PKCζ. Lamin A and β-tubulin were used as markers for the nuclear and cytoplasmic fractions, respectively. A representative experiment of three independent experiments is shown. (**F**) APPL1 associates with β-tubulin in a TRAF6-dependent manner. Cell lysates from PC-3U cells transiently transfected with control or TRAF6 siRNA were immunoprecipitated with an antibody against APPL1 and subjected to immunoblotting with a β-tubulin antibody.

To further verify the role of APPL proteins in trafficking of TβRI, we transiently co-transfected GFP-tagged APPL1 and C-terminally HA-tagged TβRI in PC-3U cells, and examined the subcellular localization of these proteins using confocal imaging. We observed that GFP-APPL1 and HA-TβRI co-localized in the nucleus in response to TGFβ, whereas the nuclear translocation of the proteins was prevented in cells treated with the PI3K inhibitor wortmannin (Figure 4A). Co-immunoprecipitation revealed that endogenous APPL1 associated with TβRI-ICD in nuclear fractions derived from PC-3U cells in a TGFβ-dependent manner; however, treatment with wortmannin inhibited the formation of a nuclear complex of APPL1 and TβRI (Figure 4B). In addition to the co-localization with APPL-positive endosomes in the cytosol (Figure 4A), TβRI was also localized in Rab5-positive endosomes (Figure 4C). TGFβ treatment induced the localization of TβRI in Rab5-positive endosomes; however, most TβRI remained close to the plasma membrane when the PC-3U cells were pretreated with wortmannin (Figure 4C). Furthermore, TβRI was detected in EEA1-positive endosomes after TGFβ treatment for 30 minutes, whereas significantly less TβRI was present in EEA1-positive endosomes after knockdown of APPL1 and APPL2 (Figure 4D). The amount of nuclear TβRI-ICD that could be detected by immunoblotting decreased after pretreatment of cells with wortmannin (Figure 4E), in line with data shown in Figure 4B. Many proteins are transported along microtubules, therefore, we investigated whether APPL1 associates with β-tubulin using co-immunoprecipitation. As shown in Figure 4F, we found that APPL1 associates with microtubules and that this interaction is promoted by TRAF6. Taken together, these observations suggest that APPL proteins are involved in trafficking of the TβRI-ICD from the endosomes to the nucleus via microtubules in a TRAF6-dependent manner, and that APPL1 maturation, shedding and nuclear accumulation are controlled by different phosphoinositides, consistent with previous observations [22, 27].

### APPL proteins promote nuclear TβRI-ICD-induced cancer invasion

**Figure 5:**
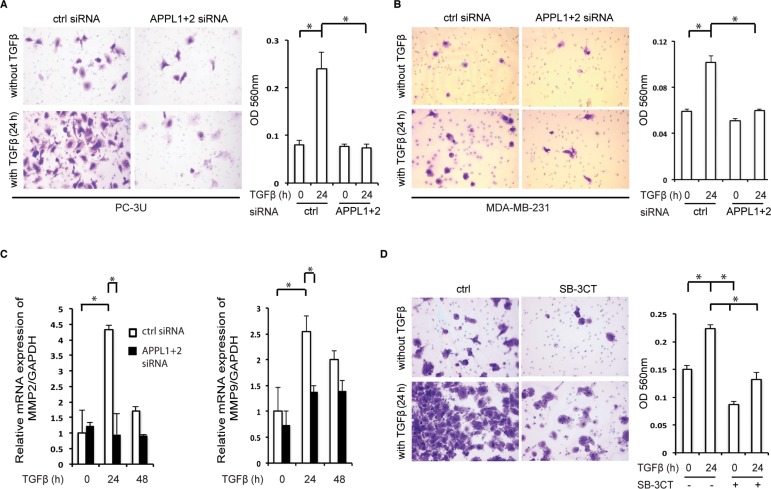
Knockdown of APPL proteins reduces TβRI-ICD-mediated cell invasion (**A**), (**B**) Invasion assays using PC-3U cells (A) and MDA-MB-231 cells (B) treated, or not, with APPL1 and APPL2 siRNA and with TGFβ. Cells were visualized by crystal violet cell staining; the panels represent the mean optical density (OD) values of invasive cells (mean ± SD, *P < 0.05; Mann-Whitney *U* test). (**C**) qRT-PCR analysis for expression of *MMP2* and *MMP9* genes was performed on mRNA extracted from PC-3U cells which had been treated with APPL1 and APPL2 siRNA and stimulated with TGFβ, as indicated. (**D**) Invasion assay using PC-3U cells that were untreated or treated with 1 μM SB-3CT and/or TGFβ (mean ± SD, *P < 0.05; Mann-Whitney *U* test).

Nuclear TβRI-ICD has been found to promote the invasion of various cancer cells [12, 13] and as APPL proteins was found to be crucial for its nuclear translocation, we investigated the role of APPL1 and APPL2 in TGFβ-induced invasiveness in cancer cells. Using a two-layer chamber, we observed TGFβ-induced invasion by the human prostate cancer PC-3U cells (Figure 5A) and breast carcinoma MDA-MB-231 cells (Figure 5B), in line with our previous reports [12, 13]. By contrast, silencing of APPL1 and APPL2 resulted in a decrease in TGFβ-induced invasion of by both these cell lines. Quantitative real-time polymerase chain reaction (qRT-PCR) analysis showed that transcription of the cancer-associated *matrix metalloprotease 2* (*MMP2*) and *MMP9* decreased after silencing of APPL1 and APPL2 (Figure 5C). Treatment of PC-3U cells with 1 μM of the MMP2 and MMP9 inhibitor SB-3CT inhibited invasion in response to TGFβ stimulation of cells (Figure 5D). These findings support the notion that APPL-promoted nuclear translocation of TβRI-ICD leads to increased expression of proteins involved in invasion, such as MMP2 and MMP9.

### APPL1 expression is higher in malignant prostate tissue than in normal prostate tissue

**Figure 6:**
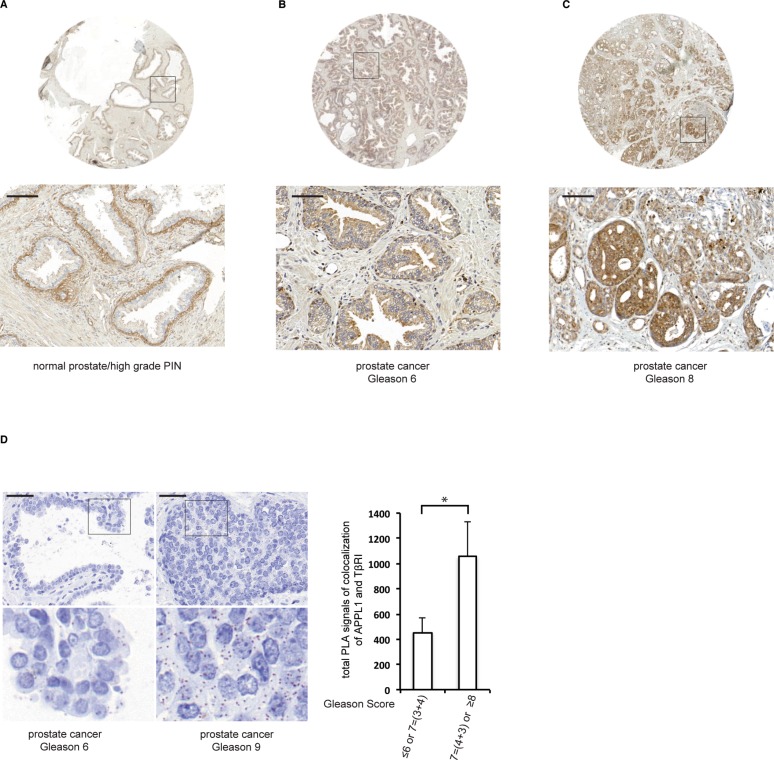
The expression of APPL1 differs in normal versus malignant prostate tissue (**A**)–(**C**) Normal prostate tissue and prostate tumour tissue samples were stained with APPL1 antibodies. Scale bar, 100 μm. (**D**) The association between APPL1 and TβRI in prostate cancer patients (brown dots) was determined by PLA; 350 cells were analyzed in each sample. Quantification shows the means ± SD of ten patients in each group. *P < 0.05; Mann-Whitney *U* test. Scale bar, 50 μm.

To further elucidate the role of APPL1 in cancer, we examined the expression and localization of APPL1 in normal and malignant prostate tissue by immunohistochemistry, as aberrant endocytosis of growth factor receptors has been found to be occur during cancer initiation and progression [35, 36]. In normal prostate tissue, APPL1 was expressed only in basal epithelial cells but not in luminal cells, whereas in malignant prostate tissues APPL1 was expressed in all cancer cells (Figure 6A–6C). APPL1 was also expressed in all epithelial cells in high-grade prostate intraepithelial neoplasia (HGPIN), suggesting that APPL1 participates in prostate cancer progression (Figure 6A). We also found that APPL1 staining, both in the cytoplasm and the nucleus, positively correlated with higher Gleason Score which is correlated to more aggressive disease (Table 1).

**Table 1:** Clinicopathologic features and APPL1 expression

Gleason score	Cytoplasmic staining			Nuclear staining	
	No.^a^	mean^b^	SE	mean^c^	SE
≤ 6 or 7 = (3 + 4)	32	2.91	0.296	0.84	0.448
7 = (4 + 3) or ≥ 8	26	4.42	0.578	2.08	0.484

a number of patients

b Cytoplasmic mean staining score; difference between groups, *P* < 0.001; Mann-Whitney *U* test

c Nuclear mean staining score; difference between groups, *P* < 0.001.

As we observed that APPL1 promotes the transport of TβRI-ICD to the nucleus of prostate cancer cells and that this is connected to invasiveness [12], we investigated whether the APPL1-TβRI-ICD complex could be found in prostate cancer tissues. Using PLA, we detected a significantly higher number of APPL1-TβRI complexes in sections from patients with aggressive prostate cancer compared with less aggressive tumours (Figure 6D).

## DISCUSSION

In this study, we have identified a role for APPL1 and APPL2 proteins in the trafficking of TβRI-ICD to the nucleus. APPL1 interacts with TβRI in a TRAF6 dependent manner and the interaction is mediated by its C-terminus. The resulting protein complex localizes to a subpopulation of early endosomes that is important for the nuclear translocation of TβRI-ICD. We also found that the TGFβ-induced invasion of cancer cells is dependent on APPL1 and APPL2 expression and that the APPL1 expression is increased in malignant prostate cancer tissues compared with normal prostate tissues. Interestingly, we also observed a greater number of APPL1-TβRI complexes in more aggressive prostate cancer tissues. Taken together, these observations suggest that the endosomal proteins APPL1 and APPL2 are important for the nuclear trafficking of TβRI-ICD, which is regulated by TRAF6 and is linked to prostate cancer progression.

APPL proteins are multifunctional adaptors proteins which associate with signalling molecules, such as PI3K, to facilitate endocytosis of various receptors [21, 22]. In this way, the APPL proteins may provide platforms for directing downstream signalling pathways. Moreover, both APPL1 and APPL2 translocate into the nucleus from the cytosol in response to EGF stimulation, to regulate cell proliferation [23, 37]. Our finding that APPL proteins interact with TβRI-ICD to promote its intracellular trafficking now expands the roles of APPL proteins to TGFβ signalling.

Nuclear TβRI-ICD, which has been observed in cancer cells both *in vivo* and *in vitro* [12], associates with the transcriptional co-regulator p300 to activate genes involved in cancer cell invasion [12]. The functional importance of nuclear TβRI-ICD is supported here by the finding that TGFβ-induced cancer cell invasion was suppressed after knockdown of APPL1 and APPL2 (Figure 5). APPL1 and APPL2 have been linked to cell survival in vertebrate development and in human glioma cells [38, 39]. APPL^−/−^ cells showed a reduced migration capacity, and knockdown of APPL2 further decreased the mobility of APPL1^−/−^ cells [40]. In tissues from prostate cancer, APPL1 expression was found to correlate with tumour malignancy (Gleason Score), indicating an important functional role for APPL1 in tumourigenesis of prostate cancer (Figure 6). Interestingly, the immunohistochemical localization of the APPL1 protein differed in healthy prostate tissue and malignant prostate tissue (Figure 6). Increased levels of APPL proteins in prostate cancer was also recently described by another group (41). It has been shown that TβRI-ICD associates with p300 and activates genes involved with tumour invasion, such as *MMP2* [12, 42]; here, we show by knockdown of APPL proteins that the nuclear TβRI-ICD-APPL protein complex regulates *MMP2* and *MMP9* expression, which could promote cancer cell invasion (Figure 5C, 5D).

The precise mechanism whereby the APPL1-TβRI-ICD complex enters the nucleus needs to be further explored, as APPL1 does not contain any obvious nuclear localization signal. A possible nuclear localization signal in APPL2 has recently been observed *in silico* [43], but its functional role has not been tested experimentally. It is known that APPL1 dissociates from early endosomes when APPL1 endosomes mature [22] and that APPL1 accumulates in the nucleus in response to EGF [23], although the molecular mechanisms behind this nuclear translocation event remain to be elucidated. TβRI-ICD formation and nuclear transport have been shown to be dependent on TRAF6, importin β1 and nucleolin [44, 45]. In this study, we found that TGFβ induces PI3K-dependent maturation of endosomes and Lys63-linked polyubiquitination of APPL1 in a TRAF6-dependent manner, promoting the nuclear translocation of APPL1 and TβRI-ICD. Future studies aiming at elucidating the detailed mechanisms for nuclear translocation of the APPL1-TβRI-ICD complex and their nuclear transcriptional targets besides *MMP2* and *MMP9*, are warranted, to better understand the protumorigenic effects of TGFβ.

In conclusion, our study has identified APPL-positive endosomes as a transient early endocytotic compartment that is crucial for trafficking of TβRI-ICD. We observed that nuclear translocation of the APPL1-TGFβRI-ICD complex correlated with an increased expression of the *MMP2* and *MMP9* genes. Moreover, a greater number of APPL1-TβRI-ICD complexes were detected in sections from aggressive human prostate cancer tissues compared with less aggressive tumours, suggesting that increased expression of APPL1 could provoke alternative TGFβ signalling routes resulting in cancer progression. Future studies in larger clinical materials will be important to evaluate the clinical value of the herein reported APPL1-TβRI-ICD complex as a potential prognostic marker in tissues from patients with cancer.

## MATERIALS AND METHODS

### Ethics statement

The investigation has been conducted in accordance with the ethical standards and according to the Declaration of Helsinki and according to national and international guidelines and has been approved by the authors' institutional review board.

### Cell culture

The PC-3U human prostatic carcinoma cell line represents a clone from the original PC-3 cell line (ATCC CRL-1435). The PC-3U cells was grown in RPMI-1640 with 10% fetal bovine serum (FBS) and 2 mM L-glutamine [46]. The human breast cancer cell line MDA-MB-231 used in this study was purchased from ATCC (ATCC CRM-HTB-26), and cells were grown in L15 media with 15% FBS and 2 mM L-glutamine. For TGFβ stimulation, TGFβ (5 ng/ml) was added in medium containing 1% FBS to cells that had been starved for 12–18 h.

### Antibodies and reagents

The following antibodies were used for immunoblotting, immunoprecipitation (IP), or immunofluorescence (IF). Antibodies against APPL1 (dilution 1:1, 000), Lamin A (1:1, 000), p-PKCζ/λ (Thr410/403, 1:1, 000), p-p38 (1:1, 000), p38 (1:1, 000) and SMAD2 (1:1, 000) were purchased from Cell Signaling; p-SMAD2 (1:500) was generated in rabbits in-house; APPL2 (1:500), TβRI (V22, 1:500) were purchased from Santa Cruz Biotechnology. The V22 antibody recognizes nuclear TβRI-ICD and its specificity was reported previously [12]; antibodies against HA (2 μg for IP), and β-actin and β-tubulin were from Roche and Sigma, respectively. APPL1 antibody for the PLA experiments was purchased from Santa Cruz Biotechnology, whereas Alexafluor 555 and 488 were from Invitrogen, and Lys63 polyubiquitin antibody from Enzo Life Science. Horseradish peroxidase-coupled secondary antibodies were purchased from Sigma, and the secondary antibody for Odyssey Clx machine was purchased from Licor Biosciences. Protein-G Sepharose and ECL Western blotting detection reagents were from GE Healthcare. Pefabloc was from Roche, and the PageRuler Prestained Protein Ladder was from Fermentas. The PI3 kinase inhibitor LY294002 (10 μM) was purchased from Calbiochem and wortmannin (100 nM) was from Sigma. The MMP2 and MMP9 inhibitor SB-3CT was purchased from Santa Cruz Biotechnology. The inhibitors were added 1 h before TGFβ stimulation, with exception of the experiment in Figure 4B, in which the cells were treated with wortmannin for 6 h before TGFβ stimulation.

### Protein analysis

For the TGFβ stimulation experiments, cells were starved for 12–18 h and then treated with TGFβ for the indicated time periods. Cells were washed twice with ice-cold phosphate-buffered saline (PBS) and lysed in ice-cold lysis buffer (150 mM NaCl, 50 mM Tris pH 8.0, 0.5% (v/v) DOC, 1% (v/v) NP-40, 10% (v/v) glycerol, 1 mM aprotinin, 1 mM Pefabloc and 2 mM sodium orthovanadate). After centrifugation, the supernatants were collected and the protein concentrations were measured using the BCA Protein Assay Kit (Thermo Scientific). Equal amounts of protein from each total cell lysate were subjected to immunoprecipitation. Immunoprecipitated proteins were resolved by SDS-PAGE using 7% or 10% Bis-Tris Precast gels (Life Technologies), Mini-PROTEAN TGX gels and Criterion TGX Precast gels (Bio-Rad), blotted onto nitrocellulose membranes, and subjected to immunoblotting as described previously [47].

### Nuclear fractionation assay

After starvation and stimulation with TGFβ1, PC-3U cells were washed twice with ice-cold PBS, and scraped in ice-cold PBS. After centrifugation for 5 min at 2000 rpm at 4°C the supernatant was discarded. The pellets were treated with ice-cold lysis buffer containing 10 mM morpholine ethanesulfonic acid (MES; pH 6.2), 10 mM NaCl, 1.5 mM MgCl_2_, 1 mM EDTA, 5 mM dithiothreitol (DTT), 1% Triton X-100, and protease inhibitors. After a second centrifugation step the supernatant containing the cytoplasmic part was removed. The remaining nuclear pellet was washed three times with ice-cold washing buffer (same as lysis buffer but without Triton X-100), and then dissolved in an ice-cold extraction buffer (25 mM Tris-HCl, pH 10.5, 1 mM EDTA, 0.5 M NaCl, 5 mM β-mercaptoethanol, 0.5% Triton X-100). Both the cytoplasmic and nuclear fractions were then centrifuged at 13,000 rpm [48].

### Immunofluorescence and microscope image acquisition

Immunofluorescence assays were performed as described previously [12, 47, 48]. In brief, cells were plated on coverslips and fixed in 4% paraformaldehyde for 30 min, treated with 0.2% Triton X-100 in PBS for 5 min at room temperature and then blocked with 10 mM glycine. Incubation with the primary antibodies was performed for 1 h at room temperature, followed by washing in PBS and then incubation with the secondary antibodies. Photomicrographs were obtained using a confocal microscope LSM 710 (Carl Zeiss) using a 63 × 1.4/NA objective lens (Carl Zeiss). The images were acquired using the Zen 2010 software in the presence of immersion oil at room temperature.

### *In situ* proximity ligation assay (PLA)

PC-3U cells were treated, or not, with TGFβ for 30 min, fixed in 4% paraformaldehyde and permeabilized in 0.2% Triton X-100. For brightfield PLA analysis, the tissue sections were pretreated with deparaffinization, retrieval and permeabilization. *In situ* proximity ligation assay was performed according to the manufacturer's instructions with the Duolink Detection Kit (Sigma). Photomicrographs for immunofluorescence were taken with an AX10 microscope (Carl Zeiss) using a 40 × 0.95/NA objective lens (Carl Zeiss). The images were acquired with Zen 2 pro software at room temperature. The camera was a Hamamatsu Digital Camera C11440. Images for brightfield were acquired with Pannoramic 250 Flash.

### *In vitro* binding assays

Purified GST-tagged APPL1 (Abnova) or recombinant GST, as a control, were immobilized on the glutathione Sepharose beads (GE Healthcare) in buffer 1 (20 mM Tris-HCl, pH 7.9, 20% glycerol, 1 mM EDTA, 5 mM MgCl_2_, 0.1% NP-40, 1 mM DTT, 0.2 mM phenylmethylsulphonyl fluoride (PMSF), 0.1 M NaCl), at 4°C overnight. The beads were washed three times with buffer 1, and one time in Buffer 2 (20 mM Tris-HCl, pH 7.9, 20% glycerol, 5 mM MgCl_2_, 5 mM GaCl_2_, 0.1% NP-40, 1 mM DTT, 0.2 mM PMSF, 0.1 M NaCl), then incubated with purified His-tagged TβRI-ICD or with lysates of HEK 293 cells transfected with HA-tagged TβRI-ICD at 4°C for 2 hours. After washing four times with buffer 1, the beads were collected and tested for the bound proteins by immunoblotting with anti-His or anti-HA antibody.

### Invasion assay

For invasion assays, we used Corning^®^ BioCoat^™^ Matrigel^®^ Invasion Chamber (Corning, Discovery Labware, Bedford, US). The collagen layer of the cell culture inserts was rehydrated by adding 500 μl of serum-free RPMI-1640 to the inner compartment, and 1 × 10^5^ cells were seeded inside of each insert in serum-free RPMI-1640 with or without TGFβ. The lower well of the invasion plate was filled with 500 μl RPMI supplemented with 10% FBS. After incubation for 24 hours, non-invasive cells were removed from the interior of the inserts. Invasive cells were photographed using an Olympus BX50 microscope with a 20 × /0.5 objective length after staining with Cell Stain Solution. The images were acquired using CellA software at room temperature, using an Olympus XC30 camera. Each insert was treated with 200 μl of Extraction Solution for 10 minutes. Optical density (OD) at 560 nm was measured in a plate reader.

### Plasmids

A plasmid encoding C-terminally HA-tagged constitutively active (ca) TβRI was a kind gift from Peter ten Dijke (Department of Molecular Cell Biology and Center for Biomedical Genetics, Leiden University Medical Center, Leiden, The Netherlands). Plasmids encoding GFP-tagged APPL1, YFP-tagged APPL1 (Δ1–272), and GFP-tagged APPL1 ΔC (Δ320–709) have been used before [23]. The GFP-TβRI plasmid was described before [12].

### siRNA transfection

On TARGET plus siRNA for APPL1 (target sequence: 5′ GGAAAUGGACAGUGAUAUA 3′), APPL2 (target sequence: 5′ AGAUCUACCUGACCGACAA 3′) siRNA, TRAF6 (target sequence: 5′ GGCCAUAGG UUCUGCAAAG 3′) and non-targeting control siRNA (sequences: 5′ UAGCGACUAAACACAUCAA 3′; 5′UAAGGCUAUGAAGAGAUAC 3′; AUGUAUUGGCCUGUAUUAG 3′; 5′ AUGAACGUGAAUUGCUCAA 3′) were obtained from Dharmacon Research (Lafayette, CO, USA). The siRNA was transfected into cells using Oligofectamine Transfection Reagent (Invitrogen) according to the manufacturer's protocol.

### Quantitative real-time PCR (qRT-PCR)

Total RNA was isolated from PC-3U cells with an RNeasy Mini Kit (Qiagen). Thermoscript RT-PCR (Invitrogen) was used for cDNA synthesis. qRT-PCR was performed with the Applied Biosystems 7900HT Fast Real-time PCR system and Power SYBR Green (Applied Biosystems) was used for detection of PCR products. The following primers were used for qRT-PCR: MMP2, forward primer (FP), AGGCCGACATCATGGTACTC, reverse primer (RP), GGTCAGTGCTGGAGAAGGTC; MMP9, FP, GCCCTTCTACGGCCACTACT, RP, TCAAAGACCGAGTCCAGCTT; glyceraldehyde-3-phosphate dehydrogenase (GAPDH), FP, TGATGACATCAAGAAGGTGGTGAAG, RP, TCCTTGGAGGCCATGTGGGCCAT. GAPDH was used as an internal control.

### Immunohistochemistry

The tissue slides were deparaffinized in dimethylbenzene, rehydrated through graded alcohols, and incubated in acidic (R&D) and Hot Rinse (Biocare Medical) for antigen retrieval in the Retriever 2100 (Proteogenix). After washing in running water, the slides were immersed in 3% hydrogen peroxide in methanol for 10 minutes to block endogenous peroxidase. After incubation in 5% goat serum, the sections were incubated overnight at 4°C in primary antibody diluted in 5% goat serum. APPL1 antibody (Cell Signaling) was used at a 1:100 dilution. After washing with PBS, the slides were incubated with secondary antibody (Envision^™^ System, Dako) for 30 minutes at room temperature. The sections were washed and developed with DAB Quanto (Thermo Scientific) under microscopic control. The reaction was stopped with tap water, and the sections were stained with hematoxylin, dehydrated, and mounted. Images were acquired with Pannoramic 250 Flash. An ethical permit to use tumour tissues for generation of tissue slides was provided by the Umeå Ethical Review Board in full agreement with the Swedish Ethical Review Act (540/03, Dnr 03–482).

### Statistical analysis

Statistical analyses were performed with SPSS 22 software for Windows. The Mann-Whitney *U* test was used to analyze differences between two independent groups. Values are expressed as the mean ± SD of at least three independent experiments if not otherwise indicated. *P* values less than 0.05 were considered statistically significant.

## SUPPLEMENTARY MATERIALS FIGURES


