# Supplementary material for: APPL proteins promote TGFβ-induced nuclear transport of the TGFβ type I receptor intracellular domain
Source: Oncotarget. 2015 Nov 18;7(1):279–92. doi: 10.18632/oncotarget.6346 (PMC4807998; doi:10.18632/oncotarget.6346)
Supplement: Supplementary file 1 [file oncotarget-07-0279-s001.pdf]

APPL proteins promote TGFβ-induced nuclear transport of the TGFβ type I receptor intracellular domain

Supplementary Materials

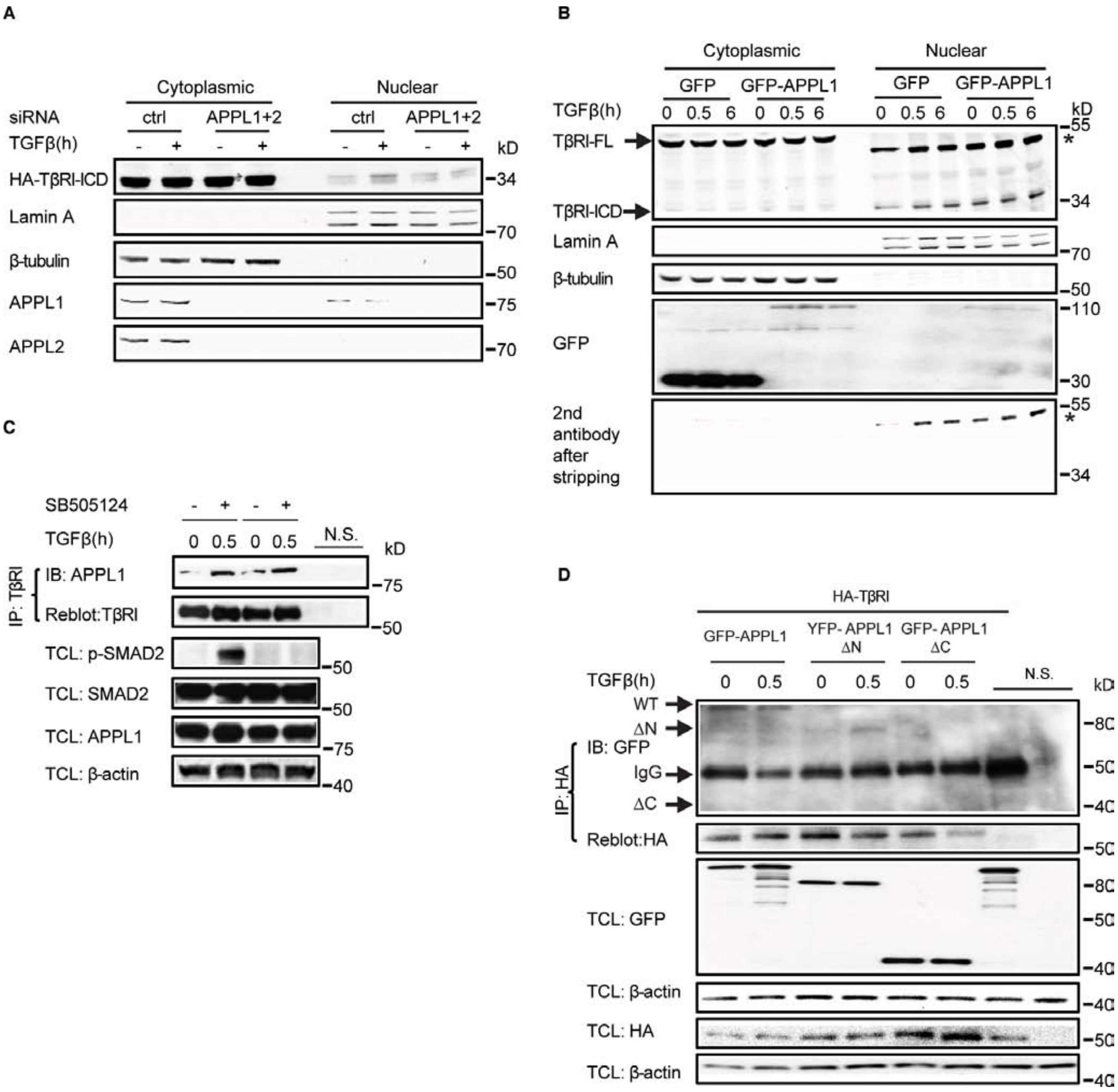

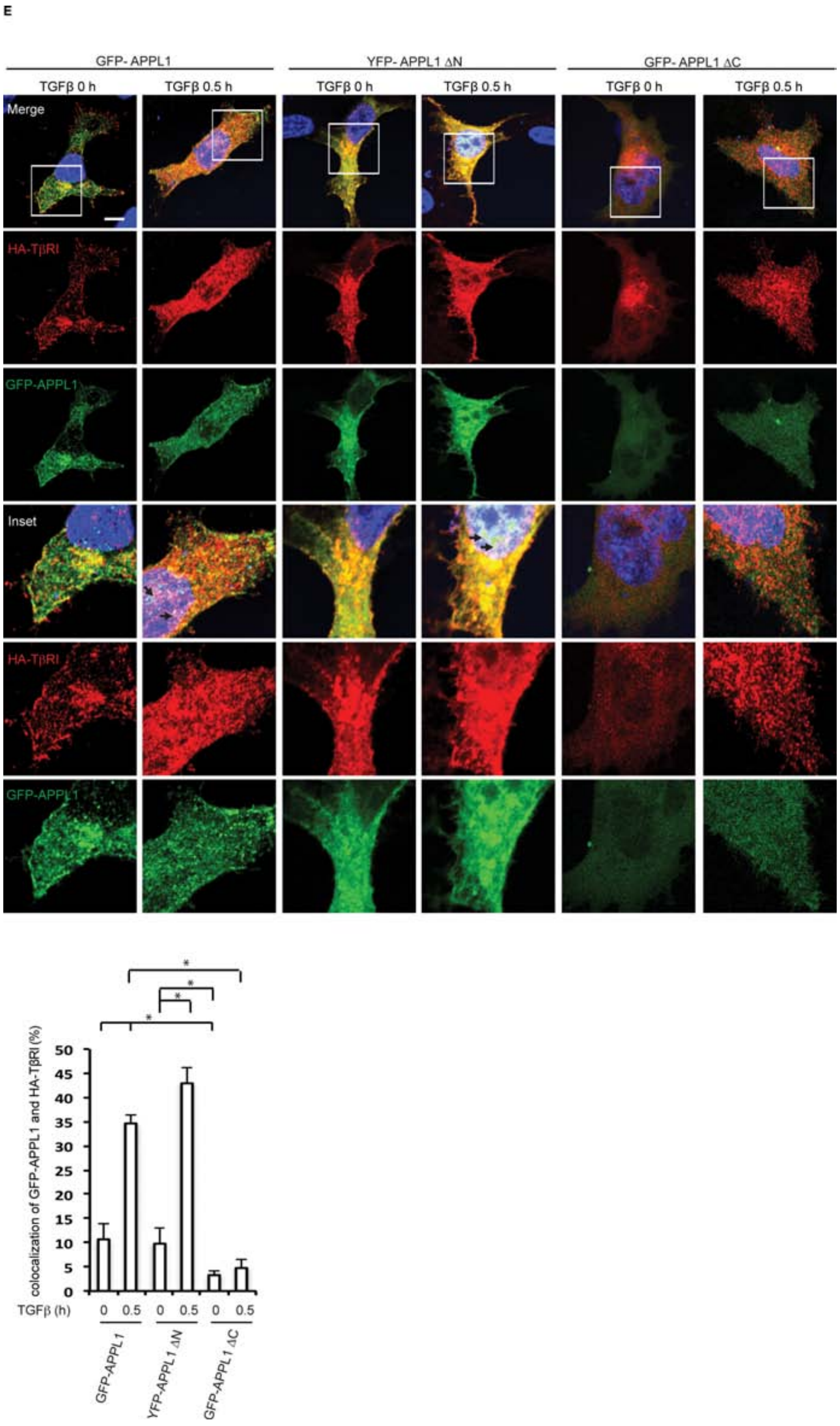

Supplementary Figure S1: APPL proteins facilitate nuclear translocation of TβRI-ICD and APPL1 associates with TβRI. (**A**) PC-3U cells transiently transfected with HA-TβRI-ICD were treated with APPL1 and APPL2 siRNA or not, and then subjected to cytoplasmic and nuclear fractionation. (**B**) PC-3U cells transiently transfected with GFP-APPL1 were fractionated into cytoplasmic and nuclear proteins. (**C**) Lysates from PC-3U cells treated, or not, with SB505124 were subjected to immunoprecipitation with a TβRI antibody (V22) followed by immunoblotting with an APPL1 antibody. (**D**) Cell lysates from PC-3U cells transiently transfected with different APPL1 domains and C-terminally linked HA-tagged TβRI (HA-TβRI), and treated as indicated, were subjected to immunoprecipitation with an antibody against HA and immunoblotted using a GFP antibody. (**E**) Immunofluorescence staining of different APPL1 domains and HA-TβRI. Their colocalization is shown as yellow dots in the merged images. Note the TGFβ-induced nuclear colocalization of the HA-TβRI and wild type (WT) or APPL1 ΔN, indicated with black arrow. Scale bar, 20 μm.

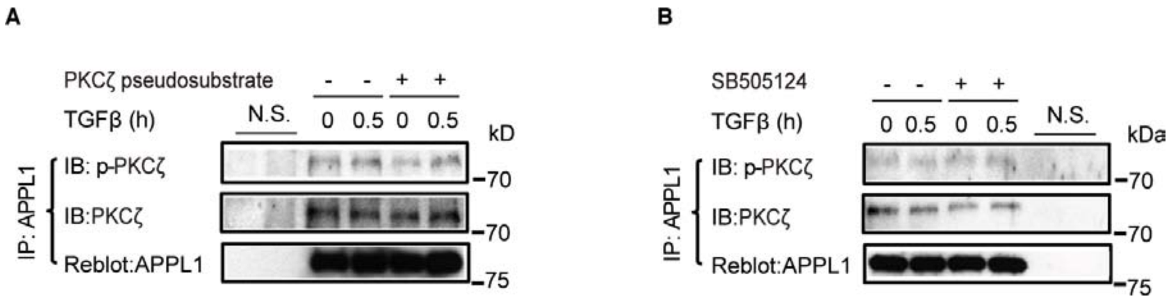

Supplementary Figure S2: APPL1 associates with PKCζ. (**A, B**) Lysates from PC-3U cells treated, or not, with PKCζ pseudosubstrate (**A**) or SB505124 (**B**) were subjected to immunoprecipitation with an APPL1 antibody followed by immunoblotting with p-PKCζ and PKCζ antibodies.
